# Volume of the rectus capitis posterior minor muscle in migraine patients: a cross-sectional structural MRI study

**DOI:** 10.1186/s10194-020-01129-y

**Published:** 2020-05-27

**Authors:** Jeppe Hvedstrup, Faisal Mohammad Amin, Anders Hougaard, Håkan Ashina, Casper Emil Christensen, Henrik Bo Wiberg Larsson, Messoud Ashina, Henrik Winther Schytz

**Affiliations:** 1 Danish Headache Center and Department of Neurology, Rigshospitalet-Glostrup, Faculty of Health and Medical Sciences, University of Copenhagen, Copenhagen, Denmark; 2 Functional Imaging Unit, Department of Clinical Physiology, Nuclear Medicine and PET, Rigshospitalet-Glostrup, Faculty of Health and Medical Sciences, University of Copenhagen, Copenhagen, Denmark

**Keywords:** Headache, Pericranial muscles, Muscle volume, RCPmi, Morphometry

## Abstract

**Background:**

Neck pain in migraine patients is very prevalent between and during migraine attacks, but the underlying mechanism behind neck pain in migraine is unknown. The neck muscle rectus capitis posterior minor muscle (RCPmi) may be important since it is connected to the occipital dura mater. In this study, we examined the RCPmi volume in migraine patients and compared with controls.

**Methods:**

We conducted a cross-sectional MRI study examining muscle volume in 40 episodic migraine patients and 40 controls in preexisting images from prior studies. Three-dimensional T1 weighted sequences were collected with a 3.0 T MRI Scanner. The volume of RCPmi was examined by manually tracing the muscle circumference with Horos medical image viewer. The observer was blinded to participant information. No information regarding neck pain status during or between migraine attacks were available.

**Results:**

The mean RCPmi volume was 1.22cm^3^ in migraine patients and 1.17cm^3^ in controls (*p* = 0.549). We found no differences in RCPmi volume on the pain side vs. the non-pain side (*p* = 0.237) in patients with unilateral migraine. There were no association between the muscle volume and years with migraine, headache or migraine frequency, age or BMI.

**Conclusions:**

We found no difference in RCPmi volume between migraine patients and controls, suggesting no structural RCPmi pathology in migraine.

## Background

Neck pain is a common feature of migraine, with a high prevalence between attacks [1] as well as during attacks [2]. This often leads migraine patients to assume cervical pathology [3], but the mechanism behind neck pain in migraine is unknown. It could be caused by convergence of nociceptive input from pericranial neck muscles and dura mater in the trigeminocervical complex [4]. In support, preclinical studies have also shown that second-order neurons localized in the cervical spinal cord are sensitized by stimulation of C-fiber afferents of the dura [5] and the neck muscles [6]. Furthermore, one study reported that stimulation of the first cervical nerve evoked frontal and periorbital pain in individuals with migraine [7]. Interestingly, the same stimulation only evoked occipital and cervical pain in migraine free individuals with chronic occipital pain [7]. Thus, nociceptive drive from upper cervical afferents might be of importance in migraine pathogenesis.

**Fig. 1:**
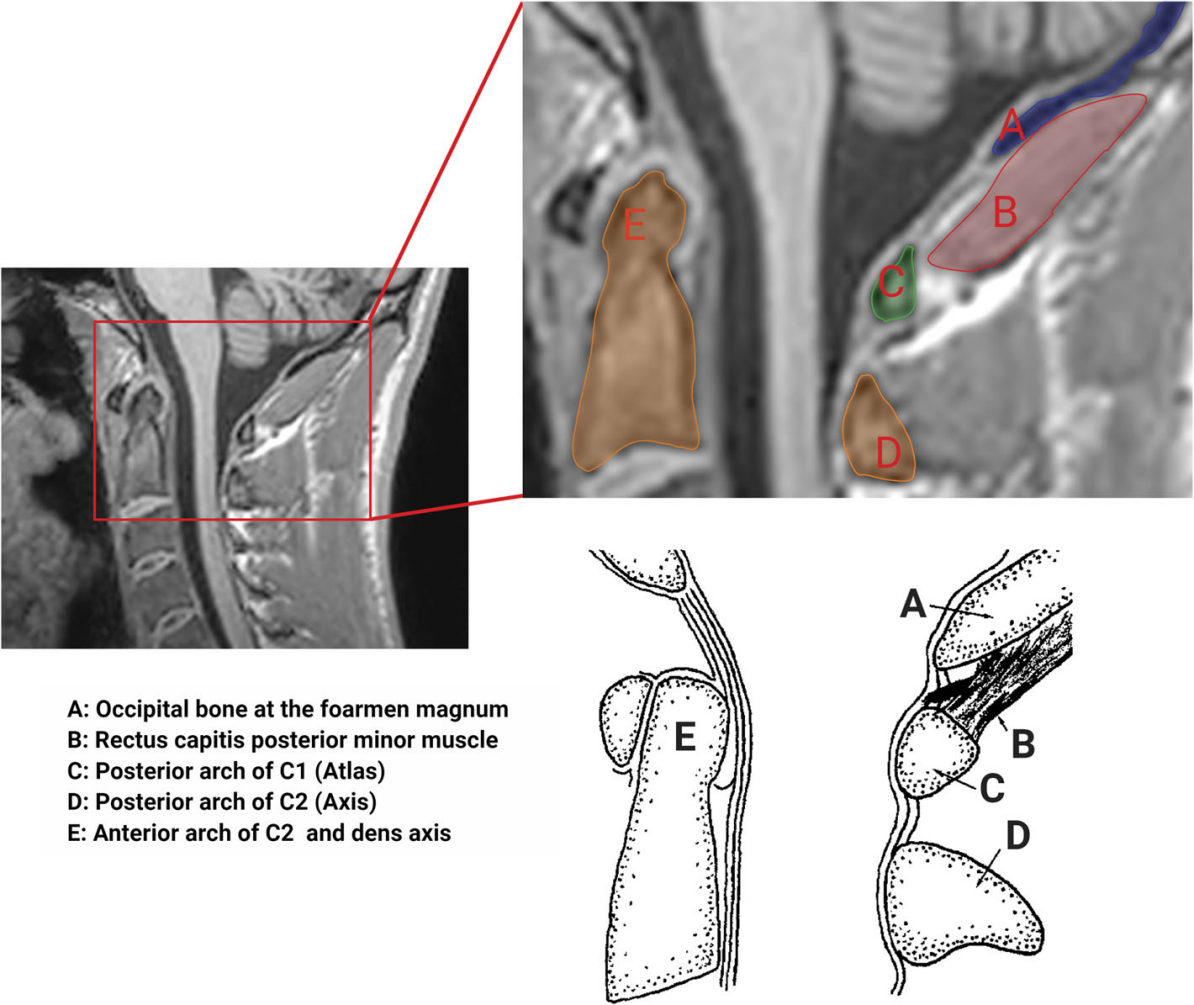
Sagittal MRI and schematics of the rectus capitis posterior minor muscle. Schematics area adapted from McPartland JM, Brodeur RR. Rectus capitis posterior minor: a small but important suboccipital muscle. *J Bodyw Mov Ther* 1999; 3: 30–35

The rectus capitis posterior minor muscle (RCPmi) is of particular interest in migraine as it is innervated by the first cervical nerve and connected to the dura via a myodural bridge [8–10] (Fig. 1). The myodural bridge is proposed to protect the dura [8] and uphold the integrity of the subarachnoid space during neck movement [11]. It is a possibility that increased activity of the RCPmi may cause hypertrophy and thereby dural tension via this myodural bridge. This may result in increased nociceptive input from upper cervical and dural afferents, which could contribute to migraine headache.

RCPmi morphometry has not previously been investigated in migraine patients, but magnetic resonance imaging (MRI) studies have demonstrated alterations in the RCPmi cross sectional area (CSA) in other headache disorders [12–14]. However, the complex RCPmi fan-shaped anatomy complicates CSA measurements, as a single CSA might not determine the exact muscle size [10]. An alternative method to assess cervical muscle size is by MRI muscle volume quantification, which recently has been validated and found reliable [15]. Hence, MRI muscle volume quantification could be a more accurate approach to determine the RCPmi muscle size.

The aim of this study was to assess the RCPmi in migraine patients compared to controls using muscle volume quantification MRI. We hypothesized that migraine patients had larger RCPmi volumes compared with controls.

## Methods

### Study population and study design

In this cross-sectional study, we examined muscle volume in 40 migraine patients and compared with 40 controls. Data from migraine patients were collected as part of a previously published project on blood-brain barrier permeability in patients with and without aura [16, 17]. These patients were compared with controls from two studies [18], (NCT03791515). Patients and controls were age-, and sex-matched using frequency matching. No data concerning muscles has been analysed from the included MRI scans prior to the current study.

This study was approved by the regional ethical committee for the Capital Region of Denmark (ID18024581). Patients with migraine with and without aura from the general population were included. Individuals were recruited through a website for recruitment of volunteers (www.forsoegsperson.dk). Controls were also recruited through posters placed at public institutions. Patients were interviewed before scanning to verify that the study criteria were met, and to establish headache frequency, migraine frequency and whether the patient had unilateral migraine. Unilateral migraine was defined as at least two-thirds of attacks with one-sided pain on the same side, in contrast to patients who had side shifting or bilateral pain during attacks.

### Inclusion and exclusion criteria for patients

Inclusion criteria for patients were age between 18 and 65 years and episodic migraine with or without aura, which was verified using the International classification of headache disorders 2nd edition [19] criteria and a thorough neurological examination. Patients were excluded if they had cardio- or cerebrovascular disease, or other primary headaches except for tension-type headache < 5 days per month. Patients with uncontrolled psychiatric disorders or contraindications to MRI were also excluded.

### Inclusion and exclusion criteria for controls

Inclusion criteria for controls were age between 18 and 65 years. Exclusion criteria were prior head trauma or whiplash injury, primary headache disorder except tension-type headache < 1 day per month, first-degree relatives with migraine or other primary headache disorders, daily medication except oral contraceptives, prior neurological or psychological disorders.

### Structural MRI data

All MRI data were collected using the same 3.0 T Philips Achieva Scanner (Philips Medical Systems, Best, The Netherlands) using a 32-element phase-array receiver head coil at Rigshospitalet Glostrup (Copenhagen, Denmark). Foam pads were placed in the head coil in both temple regions to minimise head movement. Anatomical images were acquired using a three-dimensional T1-weighted MPRAGE (magnetization-prepared rapid gradient-echo) sequence with repetition time 6.9 ms, echo time 2.78 ms (patients), 2.81–2.82 ms (controls), field-of-view 263 × 281 × 150 mm3 and matrix size 256 × 256 mm2, 137 (patients) and 145 (controls) sagittal slices, voxel size 1.1 × 1.1 × 1.1 mm3*.* Scans were performed interictally in patients, with at least 72 h since last migraine attack. Patients were instructed to call the investigator in case of migraine within 48 h after the scan.

### Magnetic resonance imaging data analysis

The images were analysed with the medical image software Horos (version 3.3.5, The Horos Project). Data analysis was performed blinded for diagnosis and clinical characteristics. Window level (WL) and window width (WW) were adjusted for optimising the visualization of the neck muscles. The midline of the RCPmi muscles was identified visually, and a region of interest (ROI) was placed by manually tracing the muscles in the sagittal plane (Fig. 1) using a pen and touch tablet (Wacom Intuos,Wacom Co, Japan). Subsequently, ROIs were placed over the muscles 10 slices to both sides; the volume of these 11 (middle + 10) slices was measured using the ROI features of the software. The volume was specified in cm^3^. The first side measured was alternately right or left from patient to patient.

#### Statistics

Skewed data are presented as median and quartiles, while normally distributed data are presented as mean and 95% confidence limits. Normal distribution of variables was tested using the Shapiro-Wilk test. Population characteristics were not normally distributed, and accordingly independent group comparisons were performed using the Mann Whitney U-test. Categorical data were analysed with Fischer’s exact test. Muscle volume was presented as the mean of right and left side. Associations were assessed using a general linear model with muscle volume as the dependent variable and the group being compared (i.e. patient/control or with/without aura) as covariate. In order to adjust for sex, it was added as a categorical covariate in the model. Paired comparisons were performed using paired T-test. If data were skewed paired comparison was performed on logarithmically transformed data. Associations between muscle volume and continuous variables were also examined using a general linear model adjusted for sex. The assumptions for the general linear models were assessed graphically using the appropriate plots. A T-test was used to compare volume in females and males. Statistics were performed using IBM SPSS Statistics 25 for Windows. The level of significance was set at two-sided 5%. The primary hypothesis was that migraine patients would have increased volume of the RCPmi compared with healthy controls. Secondary hypotheses were that we would find (i) a larger RCPmi volume on the attack side vs the non-attack side within migraine patients with unilateral migraine headache. Also, that the volume on the pain-side would be larger compared to the corresponding in matched controls; (ii) that RCPmi volume was associated with years with migraine, migraine frequency and headache frequency; (iii) that RCPmi volume in migraine patients with aura differs from migraine patients without aura. To identify possible confounders when testing muscle volume, we explored the following in both patients and controls: whether age, weight or BMI was associated with volume, and if there was a difference between sexes.

## Results

The RCPmi volume of 40 migraine patients with and without aura was evaluated using 3-T MRI. Patients were compared with 40 sex- and age-matched controls (Table 1). The median number of headache days was five, and the median migraine days was three. Seven patients (18%) had less than one migraine day per month. Nineteen (48%) patients had migraine with aura, and 25 (63%) had unilateral migraine (Table 1). No patients reported migraine within 48 h after scan.

**Table 1:** Characteristics of 40 migraine patients and 40 age-and sex-matched controls

	Controls	Migraine patients
Female, n (%)	29 (73%)	31 (78%)
Age, years	35 (28–47)	35 (28–48)
Weight, kg	67 (60–85)	70 (62–76)
Height, cm	169 (165–178)	170 (168–178)
BMI	22.9 (21.8–26.8)	22.6 (21.1–25.7)
Patients with aura, n (%)	–	19 (48%)
Patients with unilateral migraine, n (%)	–	25 (63%)
Headache days/month	–	5 (2–8)
Migraine days/month	–	3(1–5)
years with migraine	–	17(10–23)

Numerical data are shown as median and 25–75 percentiles

### RCPmi volume in migraine patients compared with controls

**Fig. 2:**
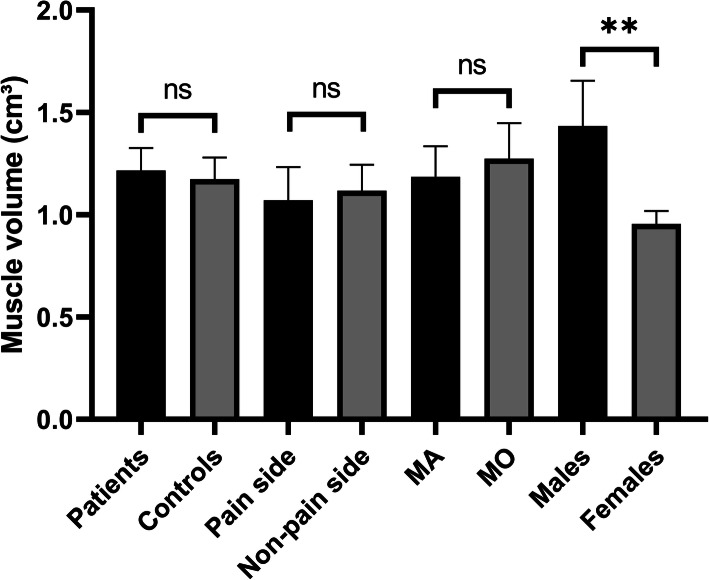
Muscle volumes of 40 migraine patients and 40 controls. Mean and 95% confidence limits are shown. MO migraine without aura, MA migraine with aura. ** indicates *p* < 0.001

The mean muscle volume in migraine patients was 1.22 cm^3^ compared with 1.17 cm^3^ in controls (*p* = 0.549) (Fig. 2). Characteristics of the groups are shown in Table 1.

### RCPmi volume in patients with unilateral migraine

In patients with unilateral migraine, the mean volume on the attack side was 1.07 cm^3^ compared with 1.12 cm^3^ on the non-attack side (*p* = 0.237) (Fig. 2). Out of the 25 patients with unilateral migraine 20 (80%) were females. We performed paired comparison of the pain side and non-pain side with the corresponding side of the sex- and age-matched individual. This did not reveal any difference on the pain side (*p* = 0.313) or non-pain side (*p* = 0.148).

### RCPmi volume in migraine with aura compared to migraine without aura

Patients with aura had a mean RCPmi volume of 1.18 cm^3^ compared with 1.27 cm^3^ in patients without aura (*p* = 0.399) (Fig. 2). We compared 19 (63% female) patients with aura with 21 patients (90% female) without aura. This was taking into consideration using the sex-adjusted general linear model.

### Associations between clinical characteristics and RCPmi volume

The RCPmi volume was not associated with selected migraine characteristics (Table 2) or population characteristics (Table 3) when adjusting for sex. Height was missing for five migraine patients.

**Table 2:** Associations between muscle volume and migraine characteristics in 40 migraine patients

	Years with migraine	Headache frequency	Migraine frequency
Association with muscle volume	−0.002 (*p* = 0.667)	0.005 (*p* = 0.736)	0.000 (*p* = 0.993)

Parameter estimates and *p-*values from the sex adjusted general linear model are shown

**Table 3:** Associations between muscle volume and characteristics in controls and migraine patients

	Age (*n* = 80)	BMI (*n* = 75)	Weight (*n* = 80)
Association with muscle volume	−0.002 (*p =* 0.484)	−0.015 (*p* = 0.144)	−0.003 (*p* = 0.389)

Parameter estimates and *p-*values from the sex adjusted general linear model are shown

### Sex stratified RCPmi volume

Overall, the RCPmi volume was smaller in women compared with men (mean 0.95 cm^3^ vs. 1.43 cm^3^, *p* < 0.001) (Fig. 2). Based on this finding, we post-hoc sex stratified the RCPmi volumes. We found that the mean RCPmi volume was 1.47 cm^3^ in male patients (*n* = 9) and 1.40 cm^3^ in male controls (*n* = 11) (*p* = 0.750), and the mean RCPmi volume was 0.97 cm^3^ in female patients (*n* = 31) and 0.94 cm^3^ in female controls (*n* = 29) (*p* = 0.599).

## Discussion

The major finding of this study was that RCPmi volume measured with MRI did not differ between episodic migraine patients and controls. Additionally, the RCPmi volume was not associated with the migraine pain side, migraine frequency or years lived with migraine. Our findings indicate that the RCPmi is not structurally altered in migraine patients.

The RCPmi has previously only been examined in non-migraine headache patients with MRI with diverging results [12–14, 20]. In 115 patients with unspecified chronic headache the RCPmi CSA was increased compared with 120 controls [12]. In contrast, another study [13] demonstrated that the mean RCPmi CSA was decreased in 15 chronic tension-type headache patients compared with 15 controls. Furthermore, active trigger points in the suboccipital muscles have been shown to be associated with smaller CSA in patients with chronic tension type headache [20]. Likewise, in 64 patients with traumatic brain injury, headache was associated with smaller CSA of the RCPmi [14]. Thus, the opposing findings suggest that RCPmi size is likely dependent on the underlying headache disorder. In addition, it is possible that some differences in RCPmi size between headache patients and controls are relatively small, and therefor require a large sample size of more than 100 patients to show differences between groups [12]. Prior studies examined patients with chronic headache which might have had an effect on the results [12, 13, 20].

Our study is the first to investigate the morphometry of the RCPmi in migraine, and there are only a few morphometry studies of neck muscles in migraine. Wanderley et al. [21] compared CSA of the longus colli muscle in 21 patients with 11 controls using ultrasound and found no differences between groups. Likewise Jull et al. [22] performed an ultrasound study and found no difference in CSA of the semispinalis capitis, the longissimus capitis or the trapezius muscles of 22 migraine patients compared with 57 controls. In contrast, an MRI study showed that the lateral pterygoid muscle CSA was increased in 20 migraine patients with comorbid temporomandibular disorder compared with 20 controls [23]. Similarly, the volumes of the medial pterygoid and masseter muscles were increased in an MRI study of ten migraine patients compared with ten controls [24]. These few studies indicate that cervical muscle size is unaffected in migraine patients, whereas masticatory muscle size may be increased.

In the present study we hypothesized that the RCPmi might have increased volume in migraine patients due to increased muscle tension. Examinations of muscle tension in migraine patients are conflicting. Thus, Pritchard et al. [25] previously showed increased muscle tension in migraine patients. This is in contrast to a recent study showing no increased muscle tension during rest or mental and physical activity in migraine patients [26]. Other studies have reported increased EMG activity of the extensors in migraine patients during active neck flexion [27, 28]. Our findings indicate no ongoing or dysfunctional activation of the RCPmi that would result in muscle hypertrophy. However, we cannot rule out that the RCPmi could be a source of nociception due to other factors such as inflammation. Furthermore, it is possible that a subgroup of migraine patients with neck pain in association with migraine attacks could have altered neck muscle volume. In support, it has previously been demonstrated that patients with ictal neck pain had increased tenderness of pericranial neck muscles compared to migraine patients without [29].

The current study has some limitations as the examined patients had a median of only three migraine days per month. Therefore, we cannot extrapolate our findings to ongoing migraine pain or to chronic migraine, where more pronounced muscle volume changes could be possible. Muscular inflammation in relatively small muscles may cause subtle volume changes, which may be difficult to identify using MRI, where small movements can influence the image. The headache and migraine frequency were established by a diagnostic interview, where a diary would have been preferred. This could have affected the association analyses. Muscle measurements were performed on existing data; hence we were not able to ask questions directed against neck symptoms or examine for myofascial pain or tenderness. Myofascial pain and neck pain in patients and headache free controls may have influenced the results. The measurements of the RCPmi included 11 slices, but in some patients a small part of the muscle may have exceeded the 11 predefined slices. This standardised method was chosen to prioritise muscle specificity over measuring the entire muscle since the lateral muscle border may be difficult to distinguish from the rectus capitis posterior major. However, we believe the chosen method is reasonable since most of the RCPmi volume is near the midline due to the fan-shaped anatomy. A strength of the current method is that scans do not need to be acquired in specialized angles, but in the sagittal plane. Thus, the method can be applied on most three-dimensional scans. Future studies should compare this new method of evaluating the RCPmi size with prior used methods or reexamine previous examined patient groups to elucidate on possible volume differences between various forms of headache.

## Conclusion

We found no structural alterations of the RCPmi in migraine patients. Further studies are warranted to explore whether the frequent neck pain is associated to functional alterations in the RCPmi. Also, if there are differences in the RCPmi volume between migraine subgroups based on neck pain symptoms, migraine localisation or frequency.

## Data Availability

The datasets used and analysed during the current study are available from the corresponding author on reasonable request.

## Abbreviations

RCPmi: Rectus capitis posterior minor muscle
CSA: Cross sectional area
MRI: Magnetic resonance imaging
WL: Window level
WW: Window width
ROI: Region of interest
BMI: Body mass index
EMG: Electromyography
